# Development of oregano essential oil nanoemulsions with considerable stability and antibacterial properties: A solution for extending the shelf life of chilled pork

**DOI:** 10.1016/j.fochx.2026.103787

**Published:** 2026-03-23

**Authors:** Siqi Zhao, Xuefei Wang, Xiaoming Guo, Chao Zhang, Qian Chen, Haotian Liu, Baohua Kong

**Affiliations:** College of Food Science, Northeast Agricultural University, Harbin, Heilongjiang 150030, China

**Keywords:** Nanoemulsion, Oregano essential oil, Nanoparticle, Interfacial interactions, Meat preservation

## Abstract

This study aimed to construct oregano essential oil nanoemulsions (OEO-NEs) with soy protein isolate/tea saponin nanoparticles (STNPs) for chilled pork preservation. Initially, the OEO-NEs with different OEO concentrations (0%, 1%, 2%, 3%, and 4%) were prepared and then characterized thoroughly. The results demonstrated that the OEO-NEs showed reduced droplet size (from 235.1 to 170.8 nm), increased surface charge (from −15.30 to −27.42 mV), and enhanced physical stability (from △BS 7.06% to △BS 0.08%) with the OEO concentration increased from 0% to 4%. Moreover, hydrogen bonding, hydrophobic interactions, and electrostatic interactions were observed between OEO and STNPs at the oil-water interface. The interfacial interactions were conducive to promoting the adsorption of STNPs, reducing the interfacial tension, and forming robust interface layers. At an OEO concentration of 4%, the interfacial tension decreased to 1.03 mN/m. The OEO-NEs exhibited increased release rate and enhanced antibacterial activities as the OEO concentration increased. OEO exhibited sustained antibacterial efficacy, as it was not fully released from the nanoemulsion systems during 15-day storage period. Moreover, the results of storage experiments revealed that 4%OEO-NE was most effective at retarding the quality deterioration of chilled pork, extending its shelf-life by 6 days. Overall, the OEO-NEs fabricated herein can ensure the safety and sensory quality of chilled pork throughout storage, offering a promising solution for active preservation in the meat industry.

## Highlights

• Hydrogen, hydrophobic, and electrostatic interactions existed between OEO and STNPs.
• The interfacial interactions promoted the adsorption of STNPs.
• 4%OEO-NE exhibited optimal droplet distribution and physical stability.
• 4%OEO-NE effectively extended the shelf life of chilled pork by 6 days.

## 1 Introduction

Chilled meat is defined as the meat product that is rapidly cooled to 0–4 °C after slaughter and then maintained at approximately 4 °C during acid removal, storage, transportation, and sale. It has gained widespread consumer preference due to its high quality and nutritional value (Liu et al., 2025). However, due to its high water activity and abundant nutrients, chilled meat is susceptible to microbial contamination, which can lead to significant economic losses and pose risks of foodborne illnesses (Lin et al., 2025). Preservatives are considered an excellent approach for extending food shelf life and improving food safety. Nowadays, with the growing health awareness among consumers, research on natural preservatives as alternatives to conventional chemical preservatives has attracted increasing attention (Kulawik et al., 2025).

Plant essential oils have been extensively studied due to their outstanding antibacterial properties, safety, and biodegradability. Oregano essential oil (OEO) is derived from the plant species *Origanum vulgare*, which primarily consists of γ-terpinene, terpinen-4-ol, and carvacrol (Juncos et al., 2025). OEO has been shown to exhibit broad-spectrum antimicrobial activity against foodborne pathogens (e.g., *Bacillus cereus*, *Salmonella enterica*, and *Escherichia coli*), spoilage microorganisms (e.g., *Pseudomonas aeruginosa*), as well as yeasts and molds. These findings highlight its significant potential as a natural preservative for meat products (Mantzourani et al., 2023). The antibacterial mechanism of OEO involves disruption of the microbial cell structure, leakage of intracellular components, and interference with the respiratory chain, which together lead to cell death (Hu et al., 2023). Nevertheless, the practical application of OEO remains constrained by its volatility under environmental stressors, such as elevated temperature, light exposure, and oxidative conditions. Moreover, the low solubility of OEO restricts its effective bioavailability. Although OEO is commonly employed to enhance food flavor (Olmedo & Grosso, 2019), its application in meat preservation frequently results in undesirable sensory outcomes. Particularly when applied to meat surfaces via soaking or spraying, the high concentration of its bioactive constituents exerts a more pronounced negative impact on sensory attributes (Zhu et al., 2025).

Oil-in-water (O/W) nanoemulsions, with the droplet sizes ranging from 20 to 500 nm, can serve as delivery systems to address the application limitations of OEO. Their uniform droplet distribution, high specific surface area and strong stability against aggregation are conducive to optimizing the water dispersibility, bioavailability, and antibacterial properties of OEO (Lu et al., 2025). Based on a previous study reported by Xing et al. (2024), the nanoemulsions showed stronger synergistic antibacterial properties compared with the cinnamon essential oil alone. In addition, Luo et al. (2025) demonstrated that the nanoemulsion system enhanced the antioxidant activity of vitamin E succinate, as evidenced by anti-radical and lipid autoxidation inhibition assays. It is important to emphasize that oxidation follows an exponential kinetic profile. Antioxidant protection agents should be administered during the early stages of oxidation to maximize inhibition of the oxidation process (Cravero et al., 2024). Overall, nanoemulsion is a preeminent encapsulation system to protect and delivery bioactive substances, enabling them to exert their biological activities effectively.

However, nanoemulsion is unstable in thermodynamics, frequently displaying Ostwald ripening, flocculation, and phase separation. Hence, emulsifiers are critical for the formation and stabilization of nanoemulsions. The emulsifiers absorb at the surfaces of oil droplets, reduce the interfacial tension, and provide strong repulsive forces between oil droplets, thereby endowing nanoemulsions with high stability (Geng et al., 2023). Recently, solid particles have been found to be superior stabilizers for constructing nanoemulsions, compared to traditional emulsifiers. These particles are irreversibly adsorbed at the oil-water interface and then form robust mechanical barriers, providing nanoemulsions with higher stability and sturdier interface structure (Cai et al., 2025). In our previous study (Zhao et al., 2024), solid nanoparticles were successfully assembled with soy protein isolate (SPI) and tea saponin (TS) through pH-driven method. Interestingly, the SPI/TS nanoparticles (STNPs) exhibited remarkable performance in constructing and stabilizing nanoemulsions containing OEO. Moreover, the nanoemulsions formed by STNPs conferred a sustained-release profile to OEO. Therefore, in this study, the STNPs were selected to fabricate OEO nanoemulsions (OEO-NEs) for chilled meat preservation.

To construct an antibacterial nanoemulsion for meat preservation, it is essential to explore the optimal concentration of the antibacterial agent, OEO, incorporated into the system. The concentration of OEO not only directly influences the antibacterial efficacy of nanoemulsions, but also significantly impacts their interfacial structure and stability. Actually, bioactive substances in the oil phase can interact with emulsifiers at oil-water interfaces, thereby affecting the characterization of nanoemulsions. According to da S. Ferreira et al. (2023), in the emulsion system, hydrogen bonds and van der Waals forces between cellulose (used as the emulsifier) and terpenes in the *Melaleuca alternifolia* essential oil were present at the oil-water interface. Tang et al. (2023) discovered that the occurrence of Schiff base reaction between chitosan and cinnamon essential oil greatly improved the stability and gel strength of the high internal phase emulsions.

Building on the above findings, we hypothesize that OEO may interact with STNPs at the oil-water interface, influencing the interfacial structure and stability of the OEO-NEs. The interfacial properties of OEO-NEs may further influence their antibacterial efficacy. The purpose of this work is to validate the aforementioned hypothesis and subsequently determine the optimal OEO concentration for preparing a nanoemulsion with robust interfacial layers and potent meat preservation capacity. At present, numerous studies have been performed to investigate the application of essential oil nanoemulsions in meat preservation. However, there has been limited exploration into the correlation between the interfacial properties of nanoemulsions and their preservation abilities.

Herein, we initially prepared OEO-NEs with different OEO concentrations (0%, 1%, 2%, 3%, 4%), and the mean droplet size, surface charge, micromorphology, and physical stability were determined to characterize OEO-NEs. Subsequently, the interfacial interactions between OEO and STNPs were investigated via interfacial tension, Fourier transform infrared (FTIR) spectra, and fluorescence spectra. To identify the practical application potential, the release and antibacterial properties of OEO-NEs were evaluated in depth. Ultimately, we comprehensively assessed the effects of OEO-NEs on the quality changes of chilled pork during storage. This work will offer a viable strategy for constructing antibacterial nanoemulsion systems for meat preservation in the future.

## 2 Materials and methods

### 2.1 Materials

SPI was purchased from Yuwang Co. (Yucheng, Shandong, China). TS, OEO, and medium chain triglyceride (MCT) oil were provided by Yuanye Bio-Technology Co., Ltd. (Shanghai, China). *Escherichia coli* (*E. coli*) (ATCC 25922) and *Staphylococcus aureus* (*S. aureus*) (ATCC 13565) were obtained from the Microbiological Culture Collection Center (Beijing, China). *Brochothrix thermosphacta* (*B. thermosphacta*) (NCBI: txid2756) was isolated from rotten pork and identified by 16 s rDNA sequencing. All other chemical reagents in this study were of analytical grade.

### 2.2 Preparation of STNP

Based on our pre*v*ious study, STNP was prepared with SPI and TS by pH-driven method (Zhao et al., 2024). Specifically, 2.0 g of SPI and 1.0 g of TS were dispersed in 150 mL of deionized water under magnetic stirring to form a homogeneous suspension (2%, *w*/*v*). The suspension was then adjusted to a pH of 12.0 using 2 M NaOH, and subsequently stirred at approximately 25 °C for 1 h. Afterwards, the pH *v*alue was regulated to 7.0 with 2 M HCl to obtain the STNP. The STNP was stored at 4 °C before measurement.

### 2.3 Characterization of STNP

#### 2.3.1 Particle size and zeta potential

Measurement of STNP particle size and zeta potential was carried out on a Mal*v*ern Zetasizer Nano-ZS90 (UK) instrument. Before the measurement, STNP suspension was diluted to 0.02% (*w*/*v*) to avoid multiple scattering effects.

#### 2.3.2 Field-emission scanning electron microscopy (FESEM)

An SN-3400 FESEM (Hitachi, Japan) was utilized to capture the micromorphology of STNP. Briefly, the STNP suspension (2%, w/v) was applied onto a cell-attached slide, and then air-dried naturally at 25 °C. Ultimately, the microstructure of the metal-sprayed sample was observed and captured by FESEM.

### 2.4 Preparation of OEO-NEs

The OEO-NEs consisted of aqueous phase (95%, *v*/v) and oil phase (5%, v/v). The STNP suspension (2%, w/v) was served as the aqueous phase of OEO-NEs. The oil phases of the nanoemulsions were prepared as follows. Briefly, MCT and OEO were mixed at the *v*olume ratio of 1:0, 4:1, 3:2, 2:3, and 1:4, respectively, and then stirred at 25 °C overnight for complete mixing. To obtain coarse emulsions, the aqueous and oil phases were homogenized by an IKA high-speed homogenizer (Germany) for 2 min at 14,000 rpm. At the pressure of 50 MPa, the coarse emulsions underwent 2 cycles in an AFM-3 high-pressure microfluidizer (Canada) to produce the OEO-NEs. The final concentration of OEO in nanoemulsion systems was 0%, 1%, 2%, 3%, and 4% (*v*/v), respectively. Notably, the OEO concentrations (0%, 1%, 2%, 3%, and 4%, v/v) were selected based on preliminary experiments. OEO concentrations below 1% showed negligible antibacterial activity, while concentrations above 4% resulted in nanoemulsion instability. Therefore, the gradient from 0% to 4% was designed to cover the effective and stable concentration window.

### 2.5 Droplet size and zeta potential of OEO-NEs

A Malvern ZetasizerNano-ZS90 instrument (UK) was used to determine the droplet size and zeta potential via dynamic light scattering. Before measurements, the OEO-NEs were diluted 100-fold with deionized water to minimize multiple light scattering effects.

### 2.6 Micromorphology of OEO-NEs

An OMX SR super-resolution microscope (GE Co, USA) was employed to observe the droplet size distribution of OEO-NEs. Briefly, each OEO-NE sample (1 mL) was stained with 20 μL of Nile red and 20 μL of Rhodamine B, followed by 30-min incubation in the dark. Then, 5 μL of stained samples were dropped on the slides. Prior to observation, the samples were stored at 4 °C.

To obtain detailed findings of the nanoemulsion interface profile, cryogenic scanning electron microscopy (cryo-SEM) was carried out using a scanning electron microscope (SN-3400, Hitachi, Japan). Specifically, the OEO-NEs were rapidly frozen with boiling liquid nitrogen. Frozen samples were then sublimated before being coated with gold. Finally, the samples were transferred to the cold module chamber for observation.

### 2.7 Backscattering light (BS) and turbiscan stability index (TSI) of OEO-NEs

The physical stability of OEO-NEs was assessed by measuring BS and TSI using a Turbiscan LAB Expert (Formulation, France). Briefly, each sample (20 mL) was transferred in a cylindrical glass cell and scanned at preset intervals by a near-infrared light beam (880 nm) from the bottom to the top for 24 h. Notably, TurbiSoft software (Formulation, France) was employed to calculate the TSI values of samples to evaluate the physical stability.

### 2.8 Interfacial tension of OEO-NEs

Based on the description of Tang et al. (2023), the interfacial tension was measured by dynamic pendant drop method through a video optical contact angle measuring instrument (OCA20, DataPhysics, Germany). A syringe containing STNP suspension was introduced into a quartz cuvette pre-filled the oil phases. Afterwards, the STNP suspension was gradually injected into oil phases with different OEO concentrations. Ultimately, the droplet images were continuously collected within 7000 s by the camera system. The interfacial tension was calculated based on the analysis of the Young Laplace equation.

### 2.9 FTIR spectroscopy

The FTIR spectra of OEO-NE (4% OEO), STNP, SPI, TS, and OEO were determined with a Nicolet IS10 FTIR spectrometer (USA) within the wavenumber range of 4000–500 cm^−1^.

### 2.10 Fluorescence spectroscopy of OEO-NEs

The fluorescence spectra of OEO-NEs were measured with the methodology of Xu et al. (2021). A F-4500 fluorescence spectrophotometer (Hitachi, Japan) was used to tested the fluorescence intensities of OEO-NEs. Before measurement, the samples were diluted 100-fold. The excitation wa*v*elength and emission slit width were 280 and 10 nm, respectively. The fluorescence intensities of samples were recorded from 280 to 350 nm.

### 2.11 Encapsulation efficiency (EE) and release profile of OEO-NEs

The EE of OEO-NEs was determined according to our previous study (Zhao et al., 2024). Ethanol, n-hexane, and OEO-NE samples were mixed in a ratio of 2:3:1 (*v*/v), followed by gentle hand-shaking for 30 s. Then, the absorbance of extracted OEO in the upper n-hexane phase was measured with a UV–Vis spectrophotometer at 255 nm. Finally, the calculation of free OEO concentration and EE was based on a standard curve generated from a series of OEO solutions. The EE was obtained from the following equation:

$$\mathrm{EE}\;\left(\%\right)=\frac{\text{Total}\;\mathrm{OEO}\;\text{amount}\;\left(g\right)-\text{Free}\;\mathrm{OEO}\;\text{amount}\;\left(g\right)}{\text{Total}\;\mathrm{OEO}\;\text{amount}\;\left(g\right)}\times 100$$

To further investigate the release profile, the OEO-NEs were stored at 4 °C and monitored every 2 days. Specifically, samples (1 mL) collected on days 1, 3, 5, 7, 9, 11, 13, and 15 were blended with ethanol and n-hexane in a 2:3:1 (v/v) ratio. After manual mixing, the absorbance of free OEO was determined at 255 nm through a UV–Vis spectrophotometer. Ultimately, the concentration of free OEO and the release percentage were obtained through the standard curve. The release percentage was calculated from the following equation:

$$\text{Release percentage}\;\left(\%\right)=\frac{\text{Free}\;\mathrm{OEO}\;\text{amount}\;\left(g\right)}{\text{Total}\;\mathrm{OEO}\;\text{amount}\;\left(g\right)}\times 100$$

### 2.12 Inhibition zone of OEO-NEs

The inhibition zone experiment was conducted to evaluate the antibacterial activity of OEO-NEs. 75 μL dilution of *E. coli*, *S. aureus*, and *B. thermosphacta* (approximately 10^7^ CFU/mL) was spread evenly on LB nutrient agar plates. Afterwards, three 10-mm sterile filter paper disks were aseptically placed on LB agar, prior to the addition of OEO-NEs (10 μL) on the filter paper. A sterile blank filter paper disk was placed on the LB nutrient agar to serve as the control. The culture media containing *E. coli* and *S. aureus* were inverted and incubated at 37 °C. For *B. thermosphacta*, the incubation temperature was set at 25 °C. Following a 24-h incubation period, the inhibition zones of OEO-NEs were examined and recorded.

### 2.13 Morphological observation of the bacteria

The micromorphology of bacteria was captured using a scanning electron microscope (SN-3400, Hitachi, Japan). In accordance with the methodology reported by Qi et al. (2022), each cell suspension of tested bacteria (1 mL, approximately 10^7^ CFU/mL) was mixed with OEO-NEs (20 μL). Then, the mixtures were incubated for 5 h. The untreated bacteria were set as the control groups. Subsequently, the incubated samples were centrifugated at 4500 *g* for 10 min to collect microorganism cells. The cells were then rinsed thrice with 0.1 M phosphate-buffered solution (PBS, pH 7.2), followed by fixation in a 2.5% glutaraldehyde solution for 12 h. Afterwards, the fixed cells were rinsed thrice with PBS and then dehydrated through graded aqueous ethanol solutions (50%, 70%, 90%, and 100%) at 15-min intervals per concentration. Finally, the samples were subject to gold sputter-coating under vacuum, and affixed to scanning electron microscopy stubs for subsequent imaging.

### 2.14 Nucleic acids and protein leakage of the bacteria

Extracellular nucleic acids and protein after OEO-NE treatments were determined with the approach of He et al. (2022). The suspensions of tested bacteria were treated with OEO-NEs, as described in section 2.13. After 5 h, processed microbial suspensions were subjected to centrifugation (10,000 *g*, 5 min) to enable the collection of the supernatant. Ultimately, the release of nucleic acids and proteins was quantified by measuring their respective absorbances at 260 nm and 280 nm.

### 2.15 Application of OEO-NEs on meat preservation

#### 2.15.1 Sample preparation

Fresh pork obtained from a local supermarket was cut into cuboids (4 × 4 × 3 cm^3^), and then dipped in 200 mL of OEO-NEs with different OEO concentrations (1%, 2%, 3%, and 4%) for 3 min. The pork samples subjected to differential treatments were placed in modified atmosphere packaging (MAP) trays and sealed under high‑oxygen MAP condition (80% O_2_, 20% CO_2_). Pork in the MAP but without soaking treatment was designated as control. Subsequently, the packaged samples were maintained at 4 °C, with analyses conducted on days 0, 3, 6, 9, 12, and 15 of the storage period.

#### 2.15.2 Measurement of pH

2 g minced pork was homogenized with 20 mL of distilled water in a test tube. The mixture was vortex-mixed for 30 s at 10-min intervals. After 30 min, the homogenate was filtered through qualitative filter paper, and the filtrate pH was measured with an electronic pH meter (Mettler Toledo, Zurich, Switzerland).

#### 2.15.3 Measurement of total *v*iable count (TVC)

The TVC of chilled pork was measured with a modified approach reported by Ma et al. (2025). Briefly, 25 g of minced sample was transferred into 225 mL of sterile saline (0.85%) and then homogenized every 10 min under aseptic conditions. After 30 min, the supernatant was diluted with sterile saline solutions. Then, the appropriate dilutions (100 μL) were evenly spread on the plate count agar. Following a 48-h incubation at 37 °C, colonies were enumerated and results were con*v*erted to log CFU/g.

#### 2.15.4 Measurement of total volatile basic nitrogen (TVB-N)

The semimicro Kjeldahl method prescribed in GB 5009.228–2016 was employed for TVB-N determination. Specifically, 10 g of minced sample was blended in 100 mL of 3% (*w*/*v*) MgO solution, followed by distilling for 5 min into 30 mL boric acid solution (2%, w/v) with a Kjeldahl distillation unit (KR-DIS-Auto, Koreatech, Korea). The resulted solution was titrated with 0.01 M HCl, and the results were reported as mg/100 g.

#### 2.15.5 Measurement of thiobarbituric acid reactive substances (TBARS)

The TBARS value was determined to evaluate the lipid oxidation of chilled pork. Minced pork samples (2 g) were transferred to test tubes, followed by sequential addition of 3 mL thiobarbituric acid solution (1%, w/v) and 17 mL trichloroacetic acid-HCl solution (2.5%, w/v). After thorough vortex homogenization, the sample was subjected to 30-min boiling water bath treatment. After cooling to approximately 25 °C, 4 mL of supernatant was extracted and mixed with 4 mL of chloroform before centrifugation (1000 *g*, 10 min). The absorbance of obtained supernatant was measured and quantified at 532 nm.

#### 2.15.6 Measurement of color

The surface color of the chilled pork was determined through a ZE-6000 colorimeter (Nippon Denshoku, Japan). The color parameters are expressed as L* (lightness), a* (redness), and b* (yellowness) units. The Euclidean net color differences (*ΔE*) were calculated through the following equation:

$$∆E=\sqrt{{\left(L*-L0*\right)}^{2}+{\left(a*-a0*\right)}^{2}+{\left(b*-b0*\right)}^{2}}$$

where *L*_0_*, *a*_0_*, and *b*_0_* are the color parameters of fresh chilled pork at day 0, and L*, a*, and b* are the color parameters of the chilled pork samples at specific storage intervals (days 3, 6, 9, 12, and 15, respectively).

### 2.16 Statistical analysis

Each experiment was conducted in triplicate, and the data were expressed as mean ± standard deviation. One-way analysis of variance (ANOVA) and Tukey's multiple comparison test were employed to evaluate the significance between the means (*P* < 0.05) in the Statistix 8.1 software package (Analytical Software, St. Paul, MN, USA).

## 3 Results and discussion

### 3.1 Characterization of STNP

**Fig. 1:**
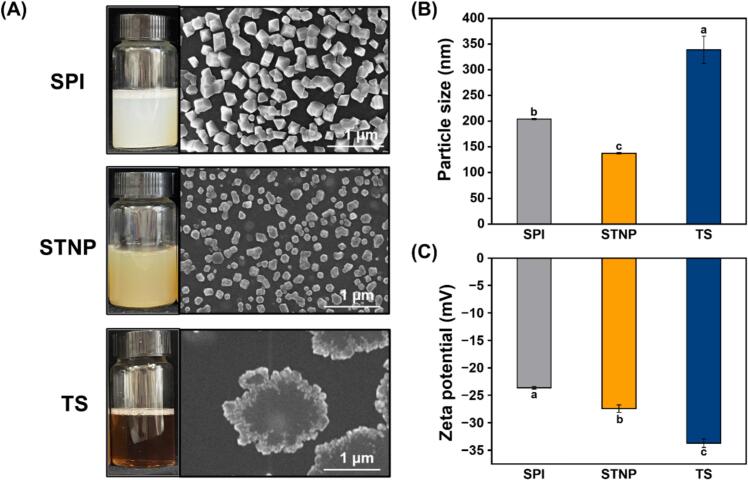
Appearance, micromorphology (A), psize (B), and zeta potential (C) of the STNP. Lowercase (a–c) letters indicate significant differences in psize and zeta potential (*P* < 0.05). SPI: soy protein isolate; TS: tea saponin; STNP: soy protein isolate/tea saponin nanoparticle.

In our previous study, we successfully assembled the nanoparticle with SPI and TS at the mass ratio of 2:1 through pH-driven method (Zhao et al., 2024). Fig. 1 depicts the appearance, micromorphology, particle size, and zeta potential of the STNP. As shown in Fig. 1A, the STNP could form a homogeneous and stable suspension in aqueous media. It can be seen from the SEM image that, the STNPs were polyhedral nanoparticles, which distributed uniformly in the vision field (Fig. 1A). Fig. 1B presents that the STNP exhibited a mean particle size of 137.8 nm, which aligned with the results of SEM analysis. Moreover, the zeta potential of the STNP was −27.46 mV, indicating the great potential in forming and stabilizing emulsion systems of STNP (Fig. 1C). Based on the above results, we constructed nanoemulsions containing OEO with different concentrations (0%, 1%, 2%, 3%, 4%) using the STNP for chilled pork preservation.

### 3.2 Micromorphology, droplet size, and zeta potential of OEO-NEs

**Fig. 2:**
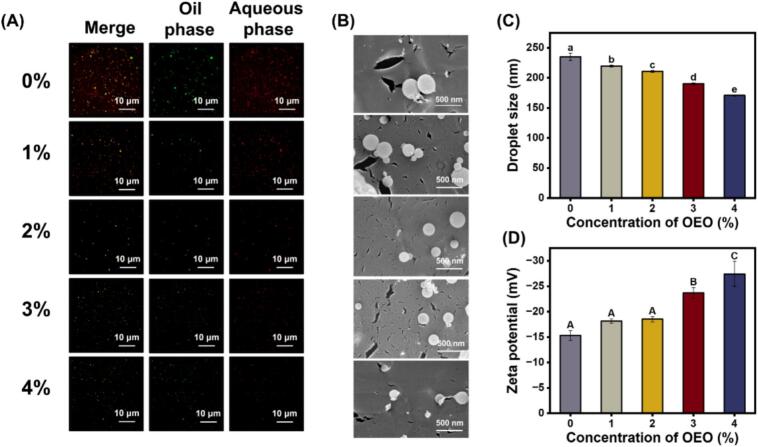
Super-resolution microscopy images (A), cryo-SEM images(B), droplet size (C), and zeta potential (D) of OEO-NEs with different OEO concentrations. Lowercase (a–e) and uppercase (A-C) letters indicate significant differences in droplet size and zeta potential, respectively (*P* < 0.05). Cryo-SEM: cryogenic scanning electron microscopy; OEO: oregano essential oil; OEO-NEs: oregano essential oil nanoemulsions.

The micromorphology of OEO-NEs was analyzed through super-resolution microscopy images (Fig. 2A) and cryo-SEM images (Fig. 2B). As shown in Fig. 2A, the green and red fluorescence represented oil droplets and STNPs, respectively. The STNPs exhibited spherical morphology with exact colocalization to oil droplets, indicating their absorption on the surface of oil droplets (Li et al., 2020). Additionally, oil droplets of each sample distributed uniformly without aggregation or flocculation, which demonstrated that the electrostatic repulsion and steric hindrance from STNPs were sufficient to resist van der Waals forces. Moreover, a progressive reduction in the droplet size of OEO-NEs was observed as the OEO concentration rose from 0% to 4%.

The cryo-SEM images exhibit the same trend. Exactly, the droplet size decreased from 235.1 nm to 170.8 nm (*P* < 0.05) (Fig. 2C). Similar results were reported by Tang et al. (2023), who discovered that the average droplet diameter of emulsions declined with the addition of cinnamon essential oil (CEO) due to the Schiff base reaction between CEO and chitosan at oil-water interfaces. In our study, OEO is a substance with a complex composition containing phenols, terpenoids, and other components, which is capable of covalent or non-covalent interactions with STNPs at surfaces of oil droplets (de Almeida et al., 2023). Hence, the increase in OEO concentration induced more compact adsorption of STNPs, hindering the droplet aggregation and thus causing the droplet size of OEO-NEs to present a downward trend. Fig. 2D illustrates that the net charge of OEO-NEs increased significantly with the increase in OEO concentration (*P* < 0.05). This phenomenon further suggests the promoted adsorption of STNPs at oil-water interfaces resulted from the interactions between OEO and STNPs. Actually, the “interfacial interaction” was our initial conjecture, with comprehensive exploration provided in subsequent sections.

### 3.3 Physical stability of OEO-NEs

**Fig. 3:**
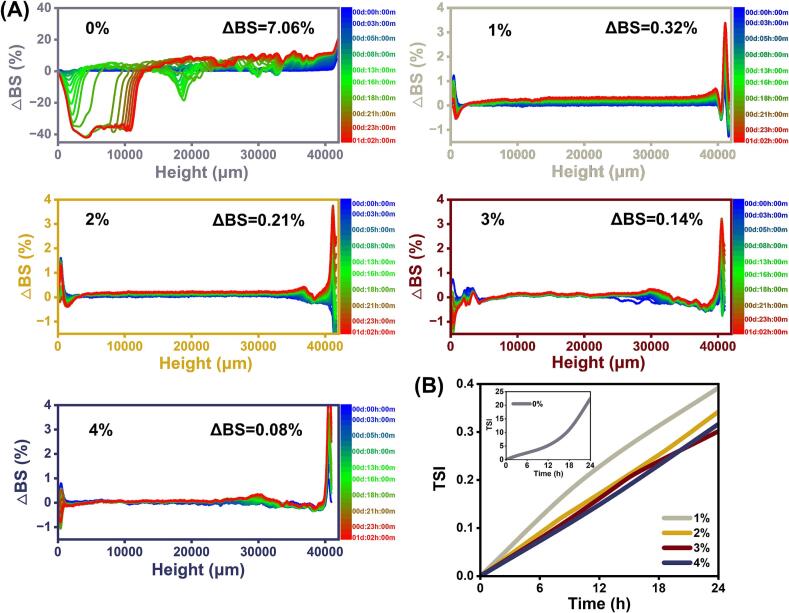
Change of backscattered light intensity in spectrum (%) (A) and Turbiscan stability index (TSI) (B) of OEO-NEs (oregano essential oil nanoemulsions).

According to Fig. 3A, each sample exhibited a positive △BS value. The BS at the bottom of OEO-NEs declined with the prolonged scanning time, which suggests that clarification and gravitational migration occurred in nanoemulsions. In contrast, the BS at the top of samples rose over time, which is due to the flotation of oil droplets (Chóez-Guaranda et al., 2025). Generally, lower △BS value indicates stronger stability of emulsion systems. When the OEO concentration was 0%, the △BS value of nanoemulsion was as high as 7.06%, exhibiting an extremely unstable state. Across the 1% to 4% OEO concentration range, the △BS gradually declined from 0.32% to 0.08%. This phenomenon can be attributed to the following reasons. When OEO was absent from the nanoemulsion system, the adsorption of STNPs was finite. Thus, the interfacial structure was relatively weak, leading to the occurrence of instability phenomena. With the addition of OEO, the interaction between OEO and STNPs was enhanced, thereby improving the adsorption of STNPs at oil-water interfaces and the formation of robust interfacial layers (Zhang et al., 2023). Hence, the OEO-NEs showed increased physical stability as the OEO concentration increasing. On the other hand, the physical stability is also related to the droplet size of OEO-NEs. Smaller oil droplets are more capable of resisting floatation and precipitation, maintaining the emulsion system stability (Gomes & Kurozawa, 2023). As the increase in OEO concentration, the interactions between OEO and STNPs were strengthened, which facilitated the capacity of STNPs to encapsulate oil droplets, resulting in the decrease in droplet size of OEO-NEs. Therefore, 4%OEO-NE demonstrated optimal physical stability.

Fig. 3B presents the TSI values of OEO-NEs. As the OEO concentration rose, the TSI value of OEO-NEs decreased progressively, indicating the enhancement of overall stability. This further suggests that the interactions between OEO and interface particles facilitated the STNP adsorption at the surfaces of oil droplets. The interactions likely involve hydrophobic interactions, hydrogen bonding, electrostatic interactions, or other covalent interactions, which will be elucidated in the subsequent investigations. Overall, the improved interfacial structure and the reduced droplet size provided high physical stability for OEO-NEs.

### 3.4 Interfacial tension analysis

**Fig. 4:**
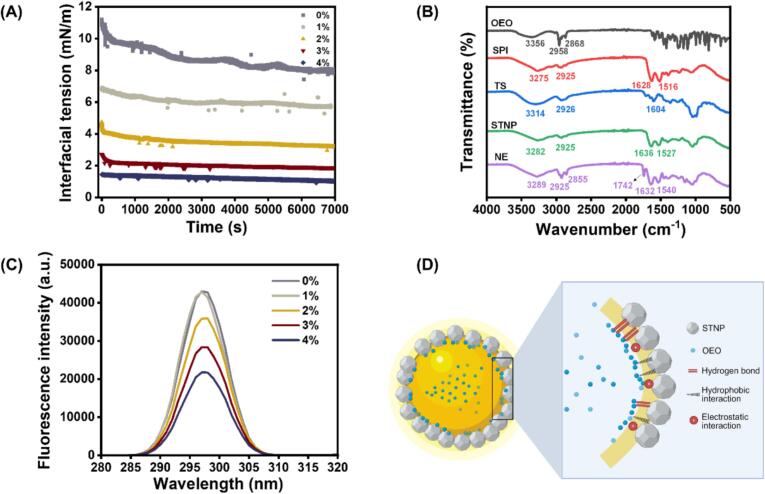
Interfacial tension of OEO-NEs (A); FTIR spectra of OEO, SPI, TS, STNP, and 4%OEO-NE (B); fluorescence intensity (C) and interfacial interactions (D) of OEO-NEs. OEO-NEs: oregano essential oil nanoemulsions; SPI: soy protein isolate; TS: tea saponin; STNP: soy protein isolate/tea saponin nanoparticle; FTIR: Fourier transform infrared spectroscopy.

During the formation of emulsions, the adsorption behavior of emulsifiers at the oil-water interface is strongly associated with the interfacial tension (Lian et al., 2023). Fig. 4A illustrates the interfacial tension of OEO-NEs containing different OEO concentrations. The interfacial tension of all samples reduced gradually as the time increased, which suggests the sustained adsorption of STNPs (Wang, Wang, et al., 2024). When the OEO concentration was 0%, the nanoemulsion exhibited the highest interfacial tension value. The interfacial tension decreased significantly with the increase in OEO concentration. When the OEO concentration reached 4%, the interfacial tension reduced to 1.03 mN/m. This is because the interactions between OEO and STNPs promoted the adsorption of STNPs, anchoring them at the oil-water interface. According to Feng et al. (2023), the eugenol and cinnamaldehyde existed in oil phase could react with the pea protein isolate at oil-water interfaces to form Schiff base and strong hydrogen bonds, improving the protein adsorption and decreasing the interfacial tension. On the other hand, the increase in OEO concentration enhanced the apolar character of the oil phase, thereby thermodynamically improving the affinity of STNPs toward the oil phase (Zou et al., 2019). Thus, both the increased apolar environment and interfacial interactions contribute synergistically to the observed reduction in interfacial tension. The results in our study were consistent with the analysis of droplet size, zeta potential, and physical stability.

### 3.5 FTIR spectroscopy analysis

To explore the interfacial interactions, FTIR spectra of OEO, SPI, TS, STNP, and 4%OEO-NE were obtained and analyzed (Fig. 4B). For SPI and TS, the peaks appearing at 3275 and 3314 cm^−1^ were related to the O—H stretching vibration. It can be observed that the O—H stretching vibration peak of STNP shifted to 3282 cm^−1^, indicating the occurrence of hydrogen bond interaction between SPI and TS (Liu et al., 2022). The peaks in the range of 1600–1700 cm^−1^ (amide I) and 1500–1600 cm^−1^ (amide II) are related to C=O stretching vibration and N − H bending vibration, respectively. According to Fig. 4B, the amide I and amide II bands of STNP shifted to 1636 and 1527 cm^−1^, respectively, compared to SPI and TS. This is because electrostatic interactions involved in the assembly of STNP. Overall, the primary driving forces underlying the assembly of STNP were hydrogen bonding and electrostatic interactions. The result is consistent with our previous study (Zhao et al., 2024).

For the spectrum of OEO, numerous high-intensity and sharp peaks were detected in the wavenumber range of 500–1700 cm^−1^. The characteristic peaks at 1521 and 1416 cm^−1^ were associated with C=C stretching vibration. The peaks appearing at 1248 and 1172 cm^−1^ could be attributed to C—O stretching vibration of phenolic hydroxyl groups. Additionally, the peaks at 862 and 810 cm^−1^ corresponded to C—H bending vibration (Wang, Waterhouse, et al., 2024). Interestingly, these characteristic peaks disappeared in the spectrum of 4%OEO-NE. This is because OEO was encapsulated in an amorphous state within the nanoemulsion system (Zhu et al., 2023). It can be found that the O—H stretching vibration peak of 4%OEO-NE appeared at 3289 cm^−1^, which demonstrates the redistribution of the hydrogen bonds between the molecules of SPI, TS, and OEO (Tang et al., 2023). Moreover, the shift of C—H stretching vibration peak indicates that OEO might be embedded in the hydrophobic cavity of SPI (Tian et al., 2023). Notably, after the introduction of OEO, C=O stretching vibration and N − H bending vibration peaks of OEO-NE shifted to 1632 and 1540 cm^−1^, respectively. This may be because OEO participated in the electrostatic interactions of SPI and TS. In summary, the non-covalent interactions between OEO and STNP included hydrogen bonding, hydrophobic interactions, and electrostatic interactions. More importantly, a new peak at 1742 cm^−1^ was observed in the spectrum of 4%OEO-NE. The formation of the new peak can be attributed to the cross-link between the phenolic hydroxyl groups in OEO and the free carboxyl group in SPI molecules (Liu et al., 2023). It was these interactions between OEO and STNP that provided OEO-NEs with stable interfacial structures.

### 3.6 Fluorescence spectroscopy analysis

To disclose the changes in interfacial protein conformation induced by OEO, the fluorescence intensity related to tryptophan groups was determined in OEO-NEs (Fig. 4C). It can be observed that the fluorescence intensity gradually decreased with the concentration of OEO increased from 0% to 4%. This is because the microenvironment of tryptophan residue in STNP was altered due to the addition of OEO. Similarly, Xu et al. (2021) explored the effect of different amounts of sweet orange essential oil (SOEO) on the fluorescence intensity correlated with tryptophan groups in the emulsions. They found that the occurrence of Schiff base reaction at the oil-water interface contributed to a fluorescence quenching effect on the interfacial proteins.

Overall, in our study, the covalent or non-covalent interactions between STNPs and OEO caused conformational changes in SPI, thereby promoting the adsorption of STNPs and the decrease in interfacial tension. Moreover, the promotional effect demonstrated a dose-dependent relationship. Specifically, the higher the OEO concentration, the more favorable it was for the occurrence of interfacial interactions. As a result, the OEO-NEs were enabled to present smaller average droplet size, increased surface charge, and enhanced physical stability.

### 3.7 Release and antibacterial activity of OEO-NEs

After investigating the stabilization mechanism of OEO-NEs, we further explored their release profile and antibacterial activities to evaluate their potential for practical applications. Since 0%OEO-NE could not demonstrate the release behavior and antibacterial ability, our subsequent exploration was exclusively focused on the 1%–4%OEO-NEs.

#### 3.7.1 EE and release profile of OEO-NEs

**Fig. 5:**
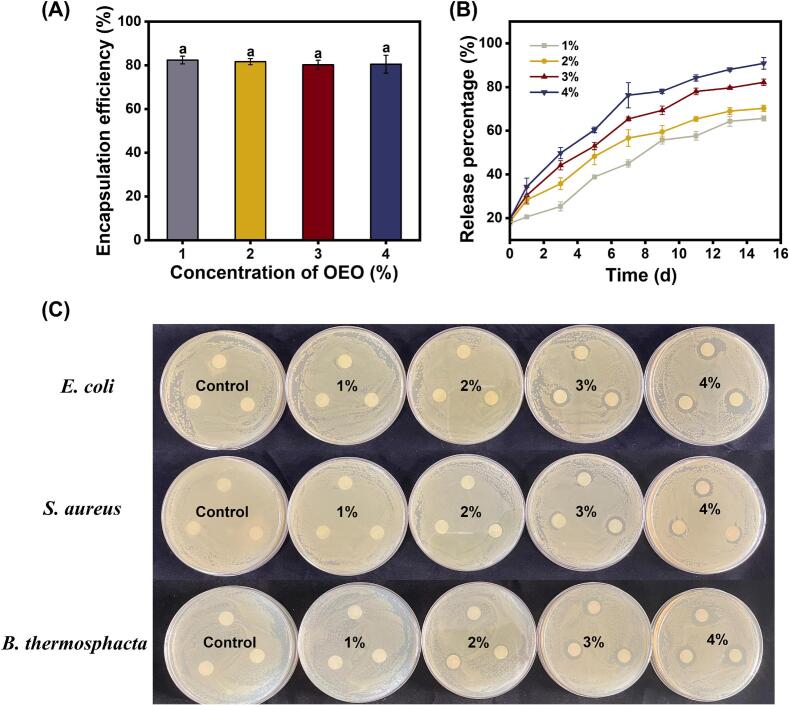
Encapsulation efficiency (EE) (A) and release percentage (B) of OEO-NEs; inhibition zone (C) OEO-NEs against *E. coli*, *S. aureus*, and *B. thermosphacta*. OEO-NEs: oregano essential oil nanoemulsions.

As shown in Fig. 5A, the EE of 1%–4% OEO-NEs was 82.42%, 81.66%, 80.27%, and 80.50%, respectively, with no statistically significant difference observed among the groups (*P* > 0.05). As reported by Liu et al. (2026), an EE greater than 80% is indicative of high encapsulation efficacy. Accordingly, the OEO-NEs prepared in this study, with EE values exceeding 80%, meet this standard.

Fig. 5B illustrates the release behavior of OEO from nanoemulsions during a 15-day storage. It can be seen that all samples presented a slow and long-term release pattern. On day 15, OEO in each nanoemulsion did not achieve complete release. The results can be explained by the following reasons. The compact and stable interface layers formed by STNPs inhibited the release of OEO. In this case, OEO released from the internal phase of nanoemulsions at a slow rate (Cai et al., 2023). On the other hand, the covalent and non-covalent interactions between OEO and STNPs immobilize OEO at the oil-water interface, thereby preventing its complete release (Nunes et al., 2024).

Furthermore, as the OEO concentration increased from 1% to 4%, the release rate and cumulative release quantity exhibited a concomitant increase, exhibiting a distinct dose-dependent effect (Fig. 5B). This is because the OEO release was based on the difference of OEO concentration between the internal phase and the external phase of nanoemulsion (Scaffaro et al., 2020). Therefore, 4%OEO-NE demonstrated the fastest release rate and the highest cumulative percentage over the same time period.

#### 3.7.2 Antibacterial activity of OEO-NEs

*E. coli* and *S. aureus* are common causative agents of foodborne illness. According to our previous study, the spoilage of pork is primarily caused by *B. thermosphacta*, which is responsible for the formation of slime and rancid odors during storage. Hence, we examined the inhibitory effects of OEO-NEs on the aforementioned three microorganisms.

Fig. 5C displayed the inhibition zone of OEO-NEs against *E. coli*, *S. aureus* and, *B. thermosphacta*. When the OEO concentration was 1%, no inhibition zones were observed in the culture medium of the three tested bacteria, indicating that 1%OEO-NE could not effectively inhibit the tested strains. When the OEO concentration increased to 2%, the nanoemulsion demonstrated low antibacterial activity. Distinct zones were observed at an OEO concentration of 3%, and the inhibition zone reached the maximum size when the OEO concentration increased to 4%. The inhibition zone of OEO-NEs was dose-dependent. As the OEO concentration increased, a greater amount of OEO diffused and released from the nanoemulsion system, thereby resulting in enhanced inhibition effect against bacteria (Yang et al., 2020). In addition, the antibacterial activity is associated with the droplet size and surface charge of nanoemulsions. He et al. (2022) reported that nanoemulsions with higher net charge are expected to exhibit a stronger interaction with bacterial membranes. Smaller droplet size of nanoemulsions is conducive to increasing the active surface area of the antibacterial agent and promoting its antibacterial effect. Therefore, in our study, 4%OEO-NE presented the highest antibacterial activity against the tested bacteria.

**Fig. 6:**
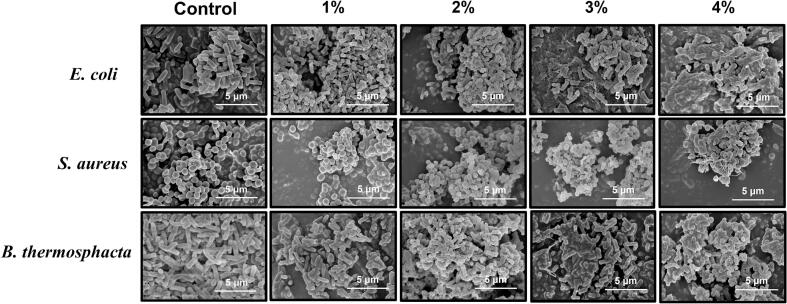
Scanning electron micrographs of *E. coli*, *S. aureus*, and *B. thermosphacta* treated by OEO-NEs (oregano essential oil nanoemulsions).

The cellular structures of *E. coli*, *S. aureus*, and *B. thermosphacta* treated by OEO-NEs were observed through a scanning electron microscope. As shown in Fig. 6, untreated cells exhibited regular shapes and smooth surfaces. However, when exposed to OEO-NEs, the cells of microorganisms were damaged to varying degrees. As OEO concentration increased from 1% to 4%, the cells displayed progressively severe structural impairments. When the OEO concentration reached 3% and 4%, the cellular morphology was distorted, accompanied by disruption of the cell membrane integrity. Moreover, the cells aggregated and adhered to each other, leading to the cellular structure unrecognizable. The results are consistent with those found by Zheng et al. (2024), who observed the micromorphology of *S. aureus*, *E. coli*, *B. cereus*, and *Sh. Boydii* treated by *Zanthoxylum bungeanum* pericarp essential oil nanoemulsions.

Generally, distortions in the cell structure of bacteria can cause leakage of intracellular contents. Thus, in the subsequent study, we quantified the nucleic acid and protein leakage from bacterial cells following treatment with OEO-NEs.

According to Table 1, with the increase in OEO concentration, a significant increase in OD_260_ and OD_280_ was observed across all three bacterial strains tested (*P* < 0.05), suggesting an elevated level of nucleic acid and protein leakage. This finding aligns with the analysis of inhibition zones and SEM images. The above results reveal that OEO-NEs were able to disrupt the integrity of bacterial membranes and then induced the release of intracellular constituents, thereby resulting in the ineluctable cell death. Additionally, the antibacterial activity of OEO-NEs depicted a strong dose dependence. Increasing the OEO concentration not only directly improved the antibacterial efficacy, but also optimized the droplet size, surface charge, and stability of OEO-NEs, enhancing their suitability for OEO delivery.

**Table 1:** Extracellular DNA and protein content of tested strains treated with OEO-NEs.

Bacterial strains	Concentration of OEO (%)	OD (260 nm)	OD (280 nm)
*E. coli*	Control	0.115 ± 0.005^Da^	0.034 ± 0.018^Da^
	1	0.229 ± 0.015^Cb^	0.156 ± 0.027^Cc^
	2	0.545 ± 0.040^Bb^	0.376 ± 0.003^Bb^
	3	0.696 ± 0.006^Ab^	0.396 ± 0.009^Bb^
	4	0.734 ± 0.002^Ab^	0.582 ± 0.044^Ab^
*S. aureus*	Control	0.130 ± 0.025^Da^	0.063 ± 0.015^Da^
	1	0.191 ± 0.015^Cc^	0.220 ± 0.002^Cb^
	2	0.420 ± 0.019^Bc^	0.245 ± 0.008^Cc^
	3	0.679 ± 0.011^Ab^	0.414 ± 0.033^Bb^
	4	0.682 ± 0.009^Ab^	0.491 ± 0.038^Ab^
*B. thermosphacta*	Control	0.109 ± 0.011^Ea^	0.065 ± 0.014^Da^
	1	0.361 ± 0.003^Da^	0.343 ± 0.004^Ca^
	2	0.730 ± 0.024^Ca^	0.729 ± 0.010^Ba^
	3	0.974 ± 0.022^Ba^	0.822 ± 0.081^Ba^
	4	1.378 ± 0.197^Aa^	1.019 ± 0.047^Aa^

Values expressed as mean ± standard deviation. A-E mean the significant differences (*P* < 0.05) between samples with the same microorganism of different OEO concentration. a-c mean the significant differences (*P* < 0.05) between samples with the same OEO concentration of different microorganism.

It is worth noting that the antibacterial capacity of OEO-NEs varied with the type of bacteria. Based on the results of inhibition zone, SEM images, and intracellular content leakage, *S. aureus* exhibited greater resistance than *E. coli* and *B. thermosphacta* to OEO-NEs. This can be attributed to the relatively thick peptidoglycan layer of *S. aureus*, which acts as a barrier, hindering the diffusion of antibacterial compounds into the cell interior (Tang et al., 2022). In contrast, *E. coli* possesses a thinner and more permeable cell envelope, making it more susceptible to the antibacterial substances in OEO-NEs (Godoy Zúniga et al., 2025). For *B. thermosphacta*, the abundance of negatively charged teichoic acid in the cell wall enhances its susceptibility to antibacterial agents through electrostatic attraction (Li et al., 2025). Overall, the OEO-NEs in our study exhibited potent antibacterial activity against all three tested strains, highlighting their strong potential for meat preservation.

### 3.8 Application of OEO-NEs on meat preservation

#### 3.8.1 pH analysis

pH is a crucial indicator of meat quality and freshness. The changes in pH of chilled pork treated by OEO-NEs are shown in Table 2. The pH of all experimental groups declined slightly during the first 3-day storage. After day 3, the pH of all samples significantly increased with the increase in storage time (*P* < 0.05), which is due to the alkaline compounds produced by meat spoilage microorganisms and endogenous enzymes (Bassey et al., 2021). The control group demonstrated the most rapid increase in pH from 3 to 15 days. By contrast, the pH of pork samples treated with OEO-NEs increased at a relatively slow rate. The increase in OEO concentration enabled OEO-NEs to more effectively retard the elevation of pH levels. At day 15, the pH of control, 1%OEO-NE, 2%OEO-NE, 3%OEO-NE, and 4%OEO-NE groups was 6.55, 6.51, 6.47, 6.33, and 6.15, respectively. Generally, a pH value exceeding 6.70 is indicative of meat spoilage (Wang et al., 2025). It seems that all pork samples in this study did not spoil throughout the entire storage period. Bassey et al. (2021) pointed out that the low pH level is attributed to the presence of CO_2_ in the packaging system. pH cannot serve as the sole criterion for assessing meat freshness. It is critical to conduct a comprehensive evaluation in combination with other indicators.

**Table 2:** Effects of OEO-NEs on the pH of chilled pork stored at 4 °C for 15 d.

OEO concentration	Storage time (d)					
	0	3	6	9	12	15
Control	5.44 ± 0.01^Ea^	5.39 ± 0.01^Ea^	5.76 ± 0.01^Da^	6.00 ± 0.03^Ca^	6.30 ± 0.01^Ba^	6.55 ± 0.01^Aa^
1%	5.53 ± 0.02^Ea^	5.45 ± 0.01^Ea^	5.74 ± 0.01^Da^	5.91 ± 0.02^Cab^	6.29 ± 0.04^Ba^	6.51 ± 0.05^Aab^
2%	5.47 ± 0.04^Ea^	5.43 ± 0.03^Ea^	5.71 ± 0.03^Dab^	5.93 ± 0.04^Cab^	6.21 ± 0.06^Ba^	6.47 ± 0.04^Aab^
3%	5.51 ± 0.01^Ea^	5.46 ± 0.03^Ea^	5.63 ± 0.04^Dbc^	5.84 ± 0.01^Cbc^	6.05 ± 0.01^Bb^	6.33 ± 0.01^Ab^
4%	5.54 ± 0.04^Ea^	5.46 ± 0.01^DEa^	5.60 ± 0.03^Dc^	5.79 ± 0.01^Cc^	6.00 ± 0.04^Bb^	6.15 ± 0.04^Ac^

Values expressed as mean ± standard deviation. Uppercase (A-F) indicate significant differences in values between time points in the same group (*P* < 0.05). Lowercase (a–e) letters indicate significant differences in values between the groups at the same time point (*P* < 0.05).

#### 3.8.2 TVC analysis

**Fig. 7:**
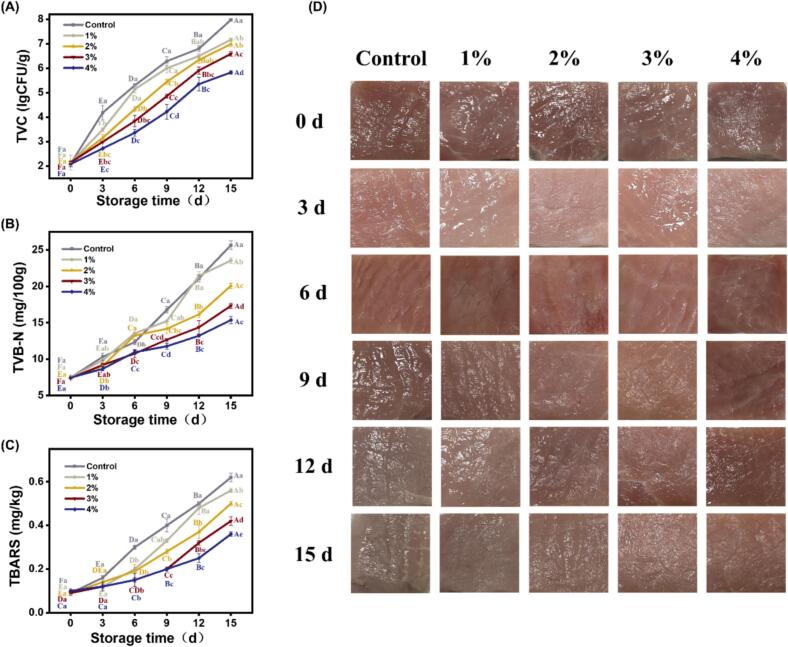
Effects of OEO-NEs on TVC (A), TVB-N (B), TBARS (C), and appearance (D) of chilled pork stored at 4 °C for 15 d. Uppercase (A-F) indicate significant differences in values between time points in the same group (P < 0.05). Lowercase (a–e) letters indicate significant differences in values between the groups at the same time point (*P* < 0.05). OEO-NEs: oregano essential oil nanoemulsions; TVC: total viable count; TVB-N: total volatile basic nitrogen; TBARS: thiobarbituric acid reactive substances.

During storage, microbial growth constitutes a critical factor contributing to meat spoilage. As shown in Fig. 7A, the TVC value of all groups increased significantly during the storage period (*P* < 0.05). At day 9, the TVC values of the control group and the 1%OEO-NE treatment group were 6.29 and 6.00 lg CFU/g, respectively, exceeding the upper limit (6.00 lg CFU/g) stipulated by Chinese Standard GB4789.2–2022. For 2% and 3%OEO-NE treatment groups, the TVC value reached the upper limit at day 12 and 15, respectively. Notably, the TVC value of chilled pork treated by 4%OEO-NE remained below 6.00 lg CFU/g throughout the entire storage period. The outstanding preservation ability of 4%OEO-NE is resulted from its high OEO concentration, high surface charge, and small droplet size. In conclusion, compared to the control group, the OEO-NE treatment groups exhibited a delayed increase in TVC values, and this delaying effect was dose-dependent.

#### 3.8.3 TVB-N analysis

According to Chinese National Standard GB5009.228–2016, the upper tolerable limit of TVB-N in pork is specified as 15.00 mg/100 g. It can be found from Fig. 7B that the TVB-N values of all groups exhibited a significant increase with prolonged storage duration (*P* < 0.05), indicating a progressive deterioration in chilled pork freshness. On day 9, the TVB-N values of the control group and the 1%OEO-NE treatment group were 16.77 and 15.24 mg/100 g, respectively, suggesting that the chilled pork had deteriorated. The TVB-N value of chilled pork treated with 2%OEO-NE exceeded the upper limit on day 12. At this point, 3%OEO-NE and 4%OEO-NE treatment groups still remained in a fresh state, and their TVB-N values surpassed the upper limit on day 15. The above results reveal that the OEO-NEs presented enhanced preservation ability as the OEO concentration increased. Similarly, a dose-dependent effect was reported by Wang et al. (2021), who found that increasing the concentration of cinnamon essential oil in nanoemulsions led to inhibited increase in TVB-N values. During storage, OEO-NEs could inhibit microorganisms and endogenous enzymes from producing biogenic amines and ammonia, thereby delaying the increase in TVB-N values of chilled pork (Wang et al., 2025).

#### 3.8.4 TBARS analysis

TBARS value is widely used to reflect lipid oxidation degree of meat, which is another important indicator to assess pork freshness (Zhu et al., 2025). A higher TBARS value indicates a more severe degree of lipid oxidation. According to Fig. 7C, the TBARS values of all groups increased significantly over the storage period (*P* < 0.05), which is attributed to the increased intensity of lipid oxidation. Compared to the control group, the OEO-NE treatment groups showed slower increase in TBARS values. Moreover, with the increase in OEO concentration, the efficacy of OEO-NEs in retarding lipid oxidation of pork depicted a dose-dependent enhancement.

#### 3.8.5 Color analysis

Color serves as a direct indicator of meat freshness and influences consumer acceptance and purchasing decisions. The changes in appearance and color parameters of chilled pork treated with OEO-NEs during storage are displayed in Fig. 7D and Table 3, respectively. As shown in Table 3, the L* values of all pork samples increased gradually during storage due to the myoglobin oxidation. Additionally, as the storage time increased, the denaturation of proteins caused them to lose the ability to bind water. Thus, large amounts of water migrated to the surface of meat, increasing the L* values (Li et al., 2022). As expected, OEO-NEs demonstrated a dose-dependent inhibition on L* value increase of chilled pork. This is because high OEO concentration is beneficial for resisting the myoglobin oxidation and maintaining the muscle structure, thereby delaying the deterioration of meat color.

**Table 3:** Effects of OEO-NEs on color characteristics of chilled pork stored at 4 °C for 15 d.

Color parameter	OEO concentration	Storage time (d)					
		0	3	6	9	12	15
L*	Control	53.81 ± 0.28^Ea^	55.84 ± 0.40^Da^	56.88 ± 0.11^Da^	58.46 ± 0.30^Ca^	60.41 ± 0.44^Ba^	62.24 ± 0.12^Aa^
	1%	53.97 ± 0.35^Ea^	55.06 ± 0.08^DEab^	55.96 ± 0.46^Dab^	57.29 ± 0.41^Cab^	58.88 ± 0.04^Bb^	61.79 ± 0.07^Aa^
	2%	53.98 ± 0.03^Ea^	55.14 ± 0.20^Dab^	55.48 ± 0.27^Dbc^	57.25 ± 0.07^Cab^	58.32 ± 0.28^Bb^	60.73 ± 0.38^Ab^
	3%	53.82 ± 0.05^Ea^	54.64 ± 0.25^DEb^	54.93 ± 0.08^Dc^	56.47 ± 0.45^Cb^	57.94 ± 0.12^Bb^	59.25 ± 0.35^Ac^
	4%	54.05 ± 0.38^Ca^	54.55 ± 0.07^Cb^	54.62 ± 0.03^Cc^	56.17 ± 0.23^Bb^	56.43 ± 0.39^Bc^	58.04 ± 0.11^Ad^
a*	Control	11.39 ± 0.05^Db^	13.52 ± 0.10^Bb^	14.74 ± 0.14^Aa^	12.49 ± 0.14^Cc^	10.48 ± 0.04^Ed^	8.98 ± 0.04^Fd^
	1%	11.54 ± 0.01^Eb^	13.82 ± 0.06^Ba^	15.01 ± 0.13^Aa^	13.48 ± 0.11^Cb^	12.75 ± 0.10^Dc^	9.87 ± 0.03^Fc^
	2%	11.47 ± 0.12^Eb^	13.49 ± 0.01^Cb^	15.12 ± 0.03^Aa^	14.06 ± 0.06^Ba^	12.95 ± 0.13^Dbc^	10.13 ± 0.15^Fc^
	3%	12.03 ± 0.04^Da^	13.86 ± 0.01^Ba^	14.91 ± 0.12^Aa^	13.96 ± 0.05^Ba^	13.24 ± 0.01^Cb^	10.78 ± 0.03^Eb^
	4%	11.32 ± 0.01^Db^	13.42 ± 0.06^Bb^	14.94 ± 0.08^Aa^	14.05 ± 0.01^BCa^	13.82 ± 0.03^Ca^	11.25 ± 0.11^Da^
b*	Control	12.03 ± 0.04^Fa^	12.56 ± 0.16^Ea^	13.85 ± 0.01^Da^	14.79 ± 0.01^Ca^	15.54 ± 0.26^Ba^	16.48 ± 0.06^Aa^
	1%	12.13 ± 0.18^Da^	12.35 ± 0.07^Da^	13.67 ± 0.17^Cab^	14.53 ± 0.07^Ba^	14.98 ± 0.03^ABb^	15.63 ± 0.32^Ab^
	2%	12.05 ± 0.06^Da^	12.42 ± 0.30^Da^	13.08 ± 0.11^Cc^	14.24 ± 0.13^Ba^	14.75 ± 0.04^ABb^	15.37 ± 0.16^Ab^
	3%	11.98 ± 0.32^Da^	12.28 ± 0.04^Da^	13.22 ± 0.08^Cbc^	13.46 ± 0.30^BCb^	14.17 ± 0.03^Bc^	15.03 ± 0.04^Ab^
	4%	12.07 ± 0.10^Ca^	12.18 ± 0.03^Ca^	12.86 ± 0.16^Bc^	13.25 ± 0.02^Bb^	13.98 ± 0.11^Ac^	14.23 ± 0.15^Ac^

Values expressed as mean ± standard deviation. Uppercase (A-F) indicate significant differences in values between time points in the same group (*P* < 0.05). Lowercase (a–d) letters indicate significant differences in values between the groups at the same time point (*P* < 0.05).

According to Table 3, the a* values of all experimental groups exhibited an increase trend from day 0 to day 6, and then decreased significantly from day 6 to day 15 (*P* < 0.05). The initial increase in the a* value was related to the rapid production of oxymyoglobin from myoglobin under high O_2_ atmosphere. The subsequent decline in the a* value was attributed to the continued oxidation of oxymyoglobin to metmyoglobin, which imparted brownish discoloration to the chilled pork. Moreover, secondary metabolites formed by microorganisms on meat surfaces could progressively reduce the a* value (Sai-Ut et al., 2026). Compared to the control group, a* values of OEO-NE treatment groups showed slower decrease rate. When the OEO concentration was 4%, the nanoemulsion could preserve the a* value of chilled pork to the greatest extent.

It can also be found from Table 3 that the b* values of all pork samples increased significantly during the storage period (*P* < 0.05), which is related to browning reactions and lipid oxidation on meat (Xiong et al., 2020). However, OEO-NE treatments effectively retarded the increase in b* values of chilled pork. This is because OEO-NEs suppressed the growth of microorganisms, reduced the production of lipase, and therefore delayed the lipid oxidation of chilled pork. Notably, with the OEO concentration increased from 1% to 4%, the delaying effect became increasingly pronounced.

The *ΔE* values of chilled pork samples are shown in Table S1. It can be observed that all treatment groups exhibited progressive increases in *ΔE* over storage time, indicating gradual color deterioration. However, OEO-NE treatments significantly retarded this process compared to the control group (*P* < 0.05). It is worth noting that 4% OEO-NE exhibited the strongest color protection during storage, with a *ΔE* of 4.54 at day 15, significantly lower than that of the control group (9.79, *P* < 0.05). Similar results were reported by Wang et al. (2021) that the *ΔE* values of chicken breasts treated with high-dose cinnamon essential oil nanoemulsions was significantly lower than the control groups during storage.

In summary, color changes occurred in chilled pork during storage period. The L* and b* values gradually increased, while the a* value first rose and then declined, indicating the meat quality deterioration. Fortunately, the OEO-NE treatments effectively mitigated discoloration and extended the shelf life of chilled pork. The preservation efficacy of OEO-NEs improved with the increase in the OEO concentration.

## 4 Conclusions

In this study, OEO-NEs containing different OEO concentrations (0%, 1%, 2%, 3%, and 4%) were successfully constructed with STNPs. Due to the interfacial covalent and non-covalent interactions between OEO and STNPs, the OEO-NEs exhibited reduced droplet size, increased surface net charge, and improved physical stability as the OEO concentration increased from 0% to 4%. Subsequently, the release profile and antibacterial activity of OEO-NEs showed dose-dependent effects, presenting the significant application potential as natural preservatives. Ultimately, the OEO-NEs combined with MAP were used to chilled pork preservation. Over the 15-day storage at 4 °C, OEO-NEs could retard the changes in pH, TVC, TVB-N, TBARS, and color properties of chilled pork. In particular, 4%OEO-NE demonstrated optimal efficacy in maintaining the pork quality and extending the shelf life by 6 days. Overall, the findings of this work suggest that OEO-NEs are promising as bioactive antibacterial agents for practical meat preservation. Future research will systematically assess the impact of OEO-NEs on meat sensory properties using electronic nose/tongue and GC–MS. Moreover, a shelf-life prediction model will be developed to enable intelligent monitoring and management of chilled meat quality and shelf-life.

## CRediT authorship contribution statement

**Siqi Zhao:** Writing – original draft, Investigation, Formal analysis, Conceptualization. **Xuefei Wang:** Software, Investigation. **Xiaoming Guo:** Visualization, Formal analysis. **Chao Zhang:** Methodology, Investigation. **Qian Chen:** Visualization, Validation, Supervision. **Haotian Liu:** Writing – review & editing, Visualization, Investigation, Funding acquisition. **Baohua Kong:** Supervision, Resources, Project administration.

## Declaration of competing interest

The authors declare that they have no known competing financial interests or personal relationships that could have appeared to influence the work reported in this paper.

## Data Availability

Data will be made available on request.

## Appendix A Supplementary data

Supplementary material Image 1
