# Supplementary material for: Development of oregano essential oil nanoemulsions with considerable stability and antibacterial properties: A solution for extending the shelf life of chilled pork
Source: Food Chem X. 2026 Mar 23;35:103787. doi: 10.1016/j.fochx.2026.103787 (PMC13054048; doi:10.1016/j.fochx.2026.103787)
Supplement: Supplementary file 1 — Supplementary material [file mmc1.docx]

Fig. S1 Schematic illustration of the STNP preparation procedure.

Table S1. Effects of OEO-NEs on *△E* values of chilled pork stored at 4 ℃ for 15 d.

| OEO concentration | Storage time (d) | | | | |
| --- | --- | --- | --- | --- | --- |
|  | 3 | 6 | 9 | 12 | 15 |
| Control | 2.99 ± 0.21^Da^ | 4.89 ± 0.54^Ca^ | 5.52 ± 0.83^Ca^ | 7.53 ± 0.29^Ba^ | 9.84 ± 0.43^Aa^ |
| 1% | 2.54 ± 0.33^Da^ | 4.29 ± 0.40^Cab^ | 4.53 ± 0.26^Cab^ | 5.80 ± 0.22^Bb^ | 8.73 ± 0.48^Aab^ |
| 2% | 2.36 ± 0.50^Da^ | 4.08 ± 0.21^Cabc^ | 4.71 ± 0.39^BCab^ | 5.32 ± 0.32^Bbc^ | 7.64 ± 0.57^Ab^ |
| 3% | 2.03 ± 0.28^Da^ | 3.33 ± 0.09^Cc^ | 3.60 ± 0.17^Cb^ | 4.82 ± 0.61^Bcd^ | 6.35 ± 0.22^Ac^ |
| 4% | 2.16 ± 0.65^Ca^ | 3.75 ± 0.37^Bbc^ | 3.65 ± 0.43^Bb^ | 3.94 ± 0.13^Bd^ | 4.54 ± 0.29^Ad^ |

Values expressed as mean ± standard deviation. Uppercase (A-D) indicate significant differences in values between time points in the same group (*P* < 0.05). Lowercase (a–d) letters indicate significant differences in values between the groups at the same time point (*P* < 0.05).
